# Supplementary material for: Exploring patient characteristics and respiratory impacts of pulmonary melioidosis: A 5-year experience from endemic region of Thailand
Source: PLoS Negl Trop Dis. 2025 Jun 25;19(6):e0013222. doi: 10.1371/journal.pntd.0013222 (PMC12212880; doi:10.1371/journal.pntd.0013222)
Supplement: Attachment — Submitted filename: PLOS review- March 2025.docx [file pntd.0013222.s002.docx]

The research article titled *Exploring Patient Characteristics and Respiratory Impacts of Pulmonary Melioidosis: A 5-Year Experience from Endemic Region of Thailand* is well-written and very informative and may be accepted after some minor corrections that needs to be addressed.

1. In Introduction section and following, after mention *Burkholderia pseudomallei* once, the name of organism may be abbreviated as *B. pseudomallei*
2. Line 73- if the study was conducted with patients why wasn’t written informed consent taken? Was there a questionnaire involved? A protocol has been submitted for the study- that have been approved by the said hospital. Please, mention whether a consent form and questionnaire were included in the protocol and why wasn’t it necessary to take written informed consent from each patient.
3. Line 86 (Data collection)- please explain briefly why MALDI-TOV-MS was used to detect bacteria if culture is the gold standard for detection of *B. pseudomallei*. If it has a better detection rate than culture (or PCR), include these data in Introduction section and justify the use of choosing this method.
4. Line 118- how was it determined that *B. pseudomallei* was a colonizer in case of the particular 2 patients- please explain.
5. Line 143- mention the full form of VFD here, as a sentence should not begin with an abbreviation.
