# Supplementary material for: Exploring patient characteristics and respiratory impacts of pulmonary melioidosis: A 5-year experience from endemic region of Thailand
Source: PLoS Negl Trop Dis. 2025 Jun 25;19(6):e0013222. doi: 10.1371/journal.pntd.0013222 (PMC12212880; doi:10.1371/journal.pntd.0013222)
Supplement: Attachment — Submitted filename: Resp impacts of pul meliodosis_response to reviewer_R1.docx [file pntd.0013222.s004.docx]

**Response to reviewer**

**Title: Exploring Patient Characteristics and Respiratory Impacts of Pulmonary Melioidosis: A 5-year Experience from Endemic Region of Thailand**

**Submission ID: PNTD-D-24-01573**

------------------------------------------------------------------------------------------------------------------------------------------

Reviewer #1:

1. In a part of the introduction: the author should review the situation of melioidosis in Thailand and the study area, as all of the study population recruited in Thailand.

**Answer**: Thank you for the suggestion. We have added a summary of the current situation of melioidosis in Thailand and the specific study area using relevant literature.

We added “A large Thai cohort study conducted over a 10-year period revealed a high incidence of melioidosis in the northeastern(1) and eastern regions(2), recognized as a hyperendemic zone, with high mortality rate of 25-40% (1, 2). The disease demonstrates increased prevalence during the rainy season.”line 50-55, page 4

2. Line 37-38: Please clarify this sentence “Due to globalization, more cases of melioidosis have been reported outside of these areas(6-8).”. How does globalization affect the increase in melioidosis cases in other areas because it is not transmitted from humans to humans? Regarding the meaning of globalization, it is related to the high movement of people from one area to another and the easy connection among them.

**Answer**: Thank you for your comment. We agree that the original statement may have been unclear. We have revised the sentence to clarify that globalization may indirectly increase melioidosis reports outside endemic areas due to increased international travel, migration, and importation of goods from endemic regions.

We revised to “Given B. pseudomallei resides in soil and water, the increasing number of international travels, migrations, and importations of goods from endemic regions contributes to its widespread dissemination into non-endemic areas(3, 4).” line 53-55 page 4.

3. Line 73: Please give a justification. Why is written informed consent not required for this study? In the methodology, the authors had to collect information (such as age, sex, weight, height, body mass index (BMI), comorbidities, history of excessive alcohol consumption, history of current smoking) for individuals who met the criteria, which included their data and risk factors. Collecting that information from patients in the study population requires consent. Is this correct?

**Answer**: According to the Declaration of Helsinki, studies involving de-identified patient data, where obtaining informed consent is impossible or impracticable, may be conducted following review and approval by a research ethics committee(declaration of Helsinki). Our study involved a retrospective chart review of anonymized data without direct patient contact. It was classified as a non-interventional, de-identified, retrospective study; a purely observational analysis and reassessment of existing database records aimed at evaluating past clinical events. The study protocol was approved by the Institutional Review Board, which granted a waiver of written informed consent., as the study posed minimal risk to participants.

We added “The study was approved and granted a waiver of written informed consent by ethics committee of Chaophraya Abhaibhubejhr hospital (IRB-BHUBEJHR-322), Prachinburi, Thailand. As this retrospective observational study involved less than minimal risk to participants, which was conducted using de-identified data (not including any live participants) and adhered to the principles of Declaration of Helsinki(5).” line 87-91, page 6.

Reviewer #2: The study seems well articulated with a good number of patients, considering the rarity of cases of melioidosis. Strong statistical analyses have been made to support the results of the research conducted. There is a minor concern about ethical issues as written informed consent have not been taken which the authors need to address.

**Answer:** According to the Declaration of Helsinki, studies involving de-identified patient data, where obtaining informed consent is impossible or impracticable, may be conducted following review and approval by a research ethics committee(declaration of Helsinki). Our study involved a retrospective chart review of anonymized data without direct patient contact. It was classified as a non-interventional, de-identified, retrospective study; a purely observational analysis and reassessment of existing database records aimed at evaluating past clinical events. The study protocol was approved by the Institutional Review Board, which granted a waiver of written informed consent., as the study posed minimal risk to participants.

We added added “The study was approved and granted a waiver of written informed consent by ethics committee of Chaophraya Abhaibhubejhr hospital (IRB-BHUBEJHR-322), Prachinburi, Thailand. As this retrospective observational study involved less than minimal risk to participants, which was conducted using de-identified data (not including any live participants) and adhered to the principles of Declaration of Helsinki(5).” line 87-91 page 6.

**Results:**

Reviewer #1:

1. Line 151-152: the authors stated that Streptococcus viridans, Klebsiella pneumoniae, and Acinetobactor baumanii were concomitantly detected in sputum cultures. Is it possible that these patients with pulmonary melioidosis were admitted to the hospital and acquired those bacteria while they were there? In the discussion section, the author may include an issue of infection prevention control in the study hospital as a possible risk factor related to their respiratory outcome.

**Answer:** Hospital-acquired pneumonia (HAP) is defined as pneumonia that develops more than 48–72 hours after hospital admission, according to the 2005 ATS guidelines for the Management of Adults with
Hospital-acquired, Ventilator-associated, and Healthcare-associated Pneumonia.(6)

In our cohort, all patients had sputum samples collected upon initial presentation to the hospital; thus, all organisms identified in this study are considered community-acquired and not associated with nosocomial transmission during hospitalization. Local infection control regulations, particularly for HAP and ventilator-associated pneumonia (VAP), are actively implemented as standard requirements for all healthcare personnel involved in patient care. These include adherence to hand hygiene protocols and the VAP care bundle recommended by Management of Adults With Hospital-acquired and Ventilator-associated Pneumonia: 2016 Clinical Practice Guidelines by the Infectious Diseases Society of America and the American Thoracic Society(7)

We added “To note, sputum samples were collected upon initial presentation at emergency room and none had predisposing risk factors of nosocomial infections; thus, all organisms identified in this study are from the community” line 177-179, page 10.

We also added “In our study, concomitant community-acquired pathogens in pulmonary infection, defined as organisms isolated from specimens collected within 48 hours of hospital admission(8), such as Streptococcus viridans, Klebsiella pneumoniae, and Acinetobacter baumannii, were also identified (56.5%). Although these organisms are generally susceptible to antibiotics commonly used in the treatment of melioidosis, such as ceftazidime and meropenem, the presence of multiple pathogens may contribute to more severe clinical presentations compared to solo pathogen. In addition, local infection control regulation, particularly for hospital acquired pneumonia and ventilator associated pneumonia (VAP), are actively implemented. These include adherence to hand hygiene protocol and VAP care bundle.(8) Further research into the impact of coinfections may offer valuable insights to enhance clinical management and guide therapeutic decisions in patients with melioidosis.” line 245-255, page 13 in discussion part.

2. Line 160-161: result of univariate logistic regression analysis showed that ARDS Pulmonary melioidosis with ARDS was associated with body mass index (BMI) < 20 Kg/m2. The author should conduct a multivariate regression to explore any confounder factors. BMI can be associated with age and certain chronic diseases, but there isn't a causal pathway that links the effects of BMI to ARDS Pulmonary melioidosis.

**Answer:** Thank you for your suggestion. We analyzed multivariate logistic regression analysis with 2 potential factors: 1. BMI < 20 kg/m^2^ and 2. Age per 5 years increase as a continuous data. We could use only 2 factors to analyze in multivariate logistic regression analysis in this study based on the rule of thumb for 10 events per one variable.(9)

We added “A multivariate logistic regression was also performed. Based on rule of thumb for 10 events per one variable(9), 2 potential factors: age per 5-year increase and BMI < 20 Kg/m^2^ were included” at method session, line 132-134, page 8.

-

We added “Multivariate logistic regression analysis revealed that BMI < 20 Kg/m² was associated with ARDS development, independent of age; odds ratio of 29.27 (95%CI 1.849 – 463.678, *p*=0.017).” at both result session of abstract and main manuscript, line 192-193, page 10-11.

The results of multivariate logistic regression analysis were added in Table 4.

We also add “Third, although a low BMI was associated with ARDS development, independent of age, the interpretation of this multivariate analysis should be approached with caution due to the small number of patients and the wide range of 95% confidence interval.” In limitation part, line 261-263, page 14.

3. Table 4: The authors used age as a category variable to compare between two groups. Is this correct? What is the reference group for age? If the author used age as a continuous variable, how were odd ratios calculated?

**Answer:** Age was used as a continuous variable. Odd ratios were calculated per unit increase. We further clarified the methoud used in the foot note of Table 4 and mentioned in the statistical analysis section on Page Line reading “Age and platelet count was calculated as continuous data.” line 132, page 8 and under table 4

Reviewer #2: yes, correct analyses have been made with appropriate tables and figures.

**Answer:** Thank you

**Conclusions:**

Reviewer #1:

1. Line 207-208: Please provide any references that present the same results of lengths of stay in survival patients with non-ARDS in pulmonary melioidosis in hospital and non-ARDS cases in community acquired pneumonia. These references could provide some assumptions for future research on this topic.

**Answer:** We have provided reference this section(10)

2. Line 217-218: The author stated that “the study relied on retrospective data collection from a single center. Thus, our data cannot be used to infer causal relationships. These two sentences may not be relevant. Please consider that the single center can affect the limitation of generalizability related to making inferences about the target population.

**Answer:** We agree that the statement may have been misleading. We have revised it to better reflect the limitation.

We replaced the previous version with “Firstly, the study was based on retrospective data collected from a single center, which may limit the generalizability of the findings to broader populations.“ line 258-260, page 14.

Reviewer #2: Yes, the authors clearly stated their shortcomings and strength which seemed appropriate. the authors also mention, how the data can help in further addressing the risks associated with this rare disease.

**Answer:** Thank you.

**PLOS review**

The research article titled *Exploring Patient Characteristics and Respiratory Impacts of Pulmonary Melioidosis: A 5-Year Experience from Endemic Region of Thailand* is well-written and very informative and may be accepted after some minor corrections that needs to be addressed.

1.In Introduction section and following, after mention *Burkholderia pseudomallei* once, the name of organism may be abbreviated as *B. pseudomallei*

**Answer:** Thank you. We have revised the manuscript to abbreviate *Burkholderia pseudomallei* as *B. pseudomallei* after its first full mention, in accordance with scientific writing standards.

2.Line 73- if the study was conducted with patients why wasn’t written informed consent taken? Was there a questionnaire involved? A protocol has been submitted for the study- that have been approved by the said hospital. Please, mention whether a consent form and questionnaire were included in the protocol and why wasn’t it necessary to take written informed consent from each patient.

**Answer:** According to the Declaration of Helsinki, studies involving de-identified patient data, where obtaining informed consent is impossible or impracticable, may be conducted following review and approval by a research ethics committee(declaration of Helsinki). Our study involved a retrospective chart review of anonymized data without direct patient contact. It was classified as a non-interventional, de-identified, retrospective study; a purely observational analysis and reassessment of existing database records aimed at evaluating past clinical events. The study protocol was approved by the Institutional Review Board, which granted a waiver of written informed consent., as the study posed minimal risk to participants.

We added “The study was approved and granted a waiver of written informed consent by ethics committee of Chaophraya Abhaibhubejhr hospital (IRB-BHUBEJHR-322), Prachinburi, Thailand. As this retrospective observational study involved less than minimal risk to participants, which was conducted using de-identified data (not including any live participants) and adhered to the principles of Declaration of Helsinki(5).” line 87-91, page 6.

3.Line 86 (Data collection)- please explain briefly why MALDI-TOV-MS was used to detec-t bacteria if culture is the gold standard for detection of *B. pseudomallei*. If it has a better detection rate than culture (or PCR), include these data in Introduction section and justify the use of choosing this method.

**Answer:** MALDI-TOV-MS is a technique used to identify organisms from bacterial colonies and is integrated into the routine bacterial culture process, unlike PCR, which is a separate method. While traditional bacterial culture and identification involve isolating the organism and examining its phenotypic characteristics, such as microscopic appearance, colony morphology, and growth on various media , MALDI-TOV-MS offers a significantly shorter turnaround time, allowing for more rapid pathogen identification.(11) This facilitates earlier, targeted treatment based on definitive pathogen identification, ultimately benefiting clinical management. Multiple studies have shown that MALDI-TOV-MS outperforms conventional techniques in identifying routine clinical bacterial isolates, particularly at the genus level. Due to its accuracy and efficiency, MALDI-TOV-MS is widely adopted in clinical microbiology laboratories worldwide, including our hospital.

We added “Gold standard of bacterial detection was identified by bacteria culture using Matrix-Assisted Laser Desorption Ionization time of Flight Mass Spectrometry (MALDI-TOV MS) technique(11). MALDI-TOV-MS is a rapid bacterial identification technique from cultured colonies, it offers faster turnaround time and more accurate identification compared to conventional method. This technique is commonly used in clinical microbiology laboratories.” line 101-106, Page 6.

4.Line 118- how was it determined that *B. pseudomallei* was a colonizer in case of the particular 2 patients- please explain.

**Answer:** These two patients were discharged home from the emergency department because mild symptom. Later, B. pseudomallei was detected in their sputum samples. The patients were called back to hospital, a review of subsequent chest radiograph was unremarkable as well as the absence of progressive respiratory symptoms without receiving any treatments. They were diagnosed with aspiration pneumonitis due to their clinical presentation and rapid recovery. B. pseudomallei sputum was noted as colonization as they were in endemic area and no risk factors for melioidosis.

We added “Four patients were excluded: age < 18 years (2 patients), *B. pseudomallei* colonization (2 patients; they were diagnosed with aspiration pneumonitis given their clinical presentations and rapid improvement of symptoms and CXR, without anti-viral or bacterial treatment.)“ line 141-144, page 8.

5.Line 143- mention the full form of VFD here, as a sentence should not begin with an abbreviation.

**Answer:** Thank you. “Ventilation free day (VFD) was first addressed under data collection”, line 97, page 6.

1) Please ensure that the CRediT author contributions listed for every co-author are completed accurately and in full.
At this stage, the following Authors/Authors require contributions: Jinjuta Ngeyvijit, Chawakorn Payackpunth, Pattawee Saengmongkonpipat, Subencha Pinsai, Antenor Rodrigues, and Vorakamol Phoophiboon. Please ensure that the full contributions of each author are acknowledged in the "Add/Edit/Remove Authors" section of our submission form.
The list of CRediT author contributions may be found here: <https://journals.plos.org/plosntds/s/authorship#loc-author-contributions>

**Answer:** Yes, we ensured that all authors have been listed for their contributions.

2) Please provide an Author Summary. This should appear in your manuscript between the Abstract (if applicable) and the Introduction, and should be 150-200 words long. The aim should be to make your findings accessible to a wide audience that includes both scientists and non-scientists. Sample summaries can be found on our website under Submission Guidelines:
<https://journals.plos.org/plosntds/s/submission-guidelines#loc-parts-of-a-submission>

**Answer:** We added **“**Melioidosis is a tropical infectious disease caused by *Burkholderia* *pseudomallei*. The diagnosis is confirmed by the growth of *B.* *pseudomallei*. Pulmonary melioidosis is the most common organ involvement of melioidosis with a high fatal rate. Acute respiratory distress syndrome (ARDS) is life-threatening lung injury characterized by poor oxygenation and non-compliant (stiff) lungs. Poor outcome has been reported in pulmonary melioidosis with ARDS. We aimed to identify clinical characteristics and potential risk factors between patients with pulmonary melioidosis with and without ARDS. Our study demonstrated that excessive alcohol consumption and a low BMI status in patients with pulmonary melioidosis could predict the development of ARDS.”

3) In the online submission form, you indicated that "The data that support the findings of this study are available on suitable requests to the corresponding authors". All PLOS journals now require all data underlying the findings described in their manuscript to be freely available to other researchers, either
- In a public repository
- Within the manuscript itself
- Uploaded as supplementary information.
This policy applies to all data except where public deposition would breach compliance with the protocol approved by your research ethics board. If your data cannot be made publicly available for ethical or legal reasons (e.g., public availability would compromise patient privacy), please explain your reasons by return email and your exemption request will be escalated to the editor for approval. Your exemption request will be handled independently and will not hold up the peer review process, but will need to be resolved should your manuscript be accepted for publication. One of the Editorial team will then be in touch if there are any issues.

**Answer:** We uploaded as supplementary information.

We revised data availability statement to “The data that support the findings of this study is uploaded in supplementary.”

 

**References**

1.	Chantratita N, Phunpang R, Yarasai A, Dulsuk A, Yimthin T, Onofrey LA, et al. Characteristics and One Year Outcomes of Melioidosis Patients in Northeastern Thailand: A Prospective, Multicenter Cohort Study. Lancet Reg Health Southeast Asia. 2023;9.

2.	Hinjoy S, Hantrakun V, Kongyu S, Kaewrakmuk J, Wangrangsimakul T, Jitsuronk S, et al. Melioidosis in Thailand: Present and Future. Trop Med Infect Dis. 2018;3(2):38.

3.	Gee JE, Bower WA, Kunkel A, Petras J, Gettings J, Bye M, et al. Multistate Outbreak of Melioidosis Associated with Imported Aromatherapy Spray. N Engl J Med. 2022;386(9):861-8.

4.	Petras JK, Elrod MG, Ty MC, Dawson P, O'Laughlin K, Gee JE, et al. Locally Acquired Melioidosis Linked to Environment - Mississippi, 2020-2023. N Engl J Med. 2023;389(25):2355-62.

5.	Association WM. World Medical Association Declaration of Helsinki: Ethical principles for medical research involving human subjects. 2013.

6.	Guidelines for the management of adults with hospital-acquired, ventilator-associated, and healthcare-associated pneumonia. Am J Respir Crit Care Med. 2005;171(4):388-416.

7.	Kalil AC, Metersky ML, Klompas M, Muscedere J, Sweeney DA, Palmer LB, et al. Management of Adults With Hospital-acquired and Ventilator-associated Pneumonia: 2016 Clinical Practice Guidelines by the Infectious Diseases Society of America and the American Thoracic Society. Clinical Infectious Diseases. 2016;63(5):e61-e111.

8.	Kalil AC, Metersky ML, Klompas M, Muscedere J, Sweeney DA, Palmer LB, et al. Management of Adults With Hospital-acquired and Ventilator-associated Pneumonia: 2016 Clinical Practice Guidelines by the Infectious Diseases Society of America and the American Thoracic Society. Clin Infect Dis. 2016;63(5):e61-e111.

9.	Ogundimu EO, Altman DG, Collins GS. Adequate sample size for developing prediction models is not simply related to events per variable. Journal of Clinical Epidemiology. 2016;76:175-82.

10.	Cilloniz C, Ferrer M, Liapikou A, Garcia-Vidal C, Gabarrus A, Ceccato A, et al. Acute respiratory distress syndrome in mechanically ventilated patients with community-acquired pneumonia. Eur Respir J. 2018;51(3).

11.	Dingle TC, Butler-Wu SM. Maldi-tof mass spectrometry for microorganism identification. Clin Lab Med. 2013;33(3):589-609.
