# Supplementary material for: Exploring patient characteristics and respiratory impacts of pulmonary melioidosis: A 5-year experience from endemic region of Thailand
Source: PLoS Negl Trop Dis. 2025 Jun 25;19(6):e0013222. doi: 10.1371/journal.pntd.0013222 (PMC12212880; doi:10.1371/journal.pntd.0013222)
Supplement: Attachment — Submitted filename: Resp impacts of pul meliodosis_response to reviewer_R2.docx [file pntd.0013222.s005.docx]

**Response to reviewer_R2**

**Title: Exploring Patient Characteristics and Respiratory Impacts of Pulmonary Melioidosis: A 5-year Experience from Endemic Region of Thailand**

**Submission ID: PNTD-D-24-01573**

Reviewer #3: Ref: PNTD-D-24-01573R1
Review Comments:

The revised manuscript has been improved, and the authors have addressed all previous reviewer concerns.
This study presents valuable clinical data from an endemic region in Thailand, contributing to the understanding of respiratory manifestations of pulmonary melioidosis and factors associated with ARDS development.

**Answer: Thank you.**

One minor issue remains:
The manuscript includes numerous technical terms and abbreviations (e.g., HFNC, MV in line 161) that are not always accompanied by their full definitions upon first use. For clarity and accessibility, especially for non-specialist readers, I recommend using full terms or ensuring that all abbreviations are fully defined when first introduced in both the abstract and main text.

**Answer: Thank you. We agreed that these 2 words be better in full term in order to promote the understanding of the readers.**

**We have changed HFNC and MV to the full term as high flow nasal cannula and mechanical ventilation thorough the manuscript.**

**We double checked all abbreviations that were fully defined at the first use as well as removed some abbreviations from the list as they have never been used more than one time in the manuscript.**
